# Molecular and physiological control of adventitious rooting in cuttings: phytohormone action meets resource allocation

**DOI:** 10.1093/aob/mcy234

**Published:** 2019-02-11

**Authors:** Uwe Druege, Alexander Hilo, José Manuel Pérez-Pérez, Yvonne Klopotek, Manuel Acosta, Fahimeh Shahinnia, Siegfried Zerche, Philipp Franken, Mohammad R Hajirezaei

**Affiliations:** 1 Leibniz Institute of Vegetable and Ornamental Crops, Erfurt, Germany; 3 Leibniz Institute of Plant Genetics and Crop Plant Research, OT Gatersleben, Stadt Seeland, Germany; 4 Instituto de Bioingeniería, Universidad Miguel Hernández de Elche, Elche, Spain; 5 Universidad de Murcia, Facultad de Biología, Campus de Espinardo, Murcia, Spain

**Keywords:** Adventitious rooting, root, wound response, auxin, plant hormones, mechanical stress, signalling, carbohydrates, mineral, source–sink, genetic, chromatin

## Abstract

**Background:**

Adventitious root (AR) formation in excised plant parts is a bottleneck for survival of isolated plant fragments. AR formation plays an important ecological role and is a critical process in cuttings for the clonal propagation of horticultural and forestry crops. Therefore, understanding the regulation of excision-induced AR formation is essential for sustainable and efficient utilization of plant genetic resources.

**Scope:**

Recent studies of plant transcriptomes, proteomes and metabolomes, and the use of mutants and transgenic lines have significantly expanded our knowledge concerning excision-induced AR formation. Here, we integrate new findings regarding AR formation in the cuttings of diverse plant species. These findings support a new system-oriented concept that the phytohormone-controlled reprogramming and differentiation of particular responsive cells in the cutting base interacts with a co-ordinated reallocation of plant resources within the whole cutting to initiate and drive excision-induced AR formation. Master control by auxin involves diverse transcription factors and mechanically sensitive microtubules, and is further linked to ethylene, jasmonates, cytokinins and strigolactones. Hormone functions seem to involve epigenetic factors and cross-talk with metabolic signals, reflecting the nutrient status of the cutting. By affecting distinct physiological units in the cutting, environmental factors such as light, nitrogen and iron modify the implementation of the genetically controlled root developmental programme.

**Conclusion:**

Despite advanced research in the last decade, important questions remain open for future investigations on excision-induced AR formation. These concern the distinct roles and interactions of certain molecular, hormonal and metabolic factors, as well as the functional equilibrium of the whole cutting in a complex environment. Starting from model plants, cell type- and phase-specific monitoring of controlling processes and modification of gene expression are promising methodologies that, however, need to be integrated into a coherent model of the whole system, before research findings can be translated to other crops.

## INTRODUCTION

Adventitious root (AR) formation is a fundamental process of root biology by which new roots are formed post-embryonically from cells of non-root tissues. Reflecting the fascinating plasticity of plants, AR formation can be observed in excised plant parts such as shoots or leaves. Excision-induced AR formation is a bottleneck for plant survival, as the development of the new root system of isolated plant fragments restores competence for water and nutrient uptake. AR formation plays an important ecological role. It contributes to the persistence of plant individuals and dynamics of plant populations (Kinsman, 1990), and can even enhance the efficiency of phytoextraction of contaminated soils (Low *et al.*, 2011). Furthermore, AR formation in technically excised shoot tips is required for the vegetative propagation of many horticultural and forestry plant species. Precise knowledge regarding the control of excision-induced AR formation provides insight into the fascinating processes underlying the regeneration ability of plants and opens up new perspectives for sustainable and efficient utilization of plant genetic resources.

AR formation in cuttings is influenced by a large set of exogenous and endogenous factors. In this review, we outline the recent progress in understanding of molecular, hormonal and metabolic control of excision-induced AR formation. Moving forward from a previous conception on the role of plant hormones with a particular focus on auxin (Druege *et al.*, 2016), the present review also considers other hormones and describes new findings on shoot tip cuttings, leaf explants and isolated thin cell layers (TCLs) regarding the function of molecular factors and epigenetics in AR formation. Further involving the source–sink network of nutrients and metabolites in cuttings *per se* and being linked to the hormonal pathways, we provide a new holistic, system-oriented view on AR formation in cuttings as determined by genetic, epigenetic and environmental factors at stock plant and cutting level. Finally we will discuss the challenges and outlook of future research.

## EXCISION-INDUCED AR FORMATION IN SHOOT TIP CUTTINGS: PHYSIOLOGY AND DEVELOPMENTAL PHASES

Shoot tip cuttings are generated by the excision of young, mostly axillary shoots from stock plants and consist of a leafy stem with a terminal shoot apex and at least one fully developed leaf. Two stimulating principles may contribute to excision-induced AR formation: wounding at the cutting site and physical isolation from the resource and signalling network of the stock plant. Deprivation of the root system interrupts the root-sourced delivery of water, nutrients and plant hormones such as cytokinins (CKs), concurrently leading to the accumulation of substances that are usually transported downwards, such as auxin, above the cutting site.

In response to excision, a new developmental programme is initiated in particular responsive cells in the stem base near the wound, ultimately leading to the regeneration of a new root system. Depending on the plant and type of explant, diverse cell types, here referred to as AR source cells, may generate ARs (Altamura, 1996). AR formation in stem tissues has repeatedly been observed to originate in the cambium or vascular tissues, where it involves sequential phases (da Costa *et al.*, 2013). The initial phase, generally referred to as the induction phase, is characterized as an anatomical lag phase devoid of cellular changes, during which the initial cell reprogramming occurs. If the AR source cells are root-competent already, they can be fate-converted directly to AR root founder cells by a root-inducing signal. However, often the cells from which AR starts first have to acquire root competence involving dedifferentiation before they can respond to a root-inducing signal (Altamura, 1996; Ikeuchi *et al.*, 2016). After determination of AR founder cells, the initiation of ARs starts with qualitative changes in cell structures, followed by cell division and differentiation of the new cell clusters into dome-shaped root primordia. The final expression phase begins with the differentiation of primordia into the complete root body, with differentiated vascular bundles connected to the vascular cylinder of the stem, followed by the emergence of roots.

## PLANT HORMONE HOMEOSTASIS, SIGNALLING AND FUNCTION IN EXCISION-INDUCED AR FORMATION

### Auxin as key factor

Cutting excision from the donor plant greatly modifies plant hormone homeostasis in the isolated shoot. It is widely accepted that auxin is an effective inducer of AR formation (Pacurar *et al.*, 2014). Polar auxin transport (PAT) plays a crucial role in controlling the level of indole-3-acetic acid (IAA), which is the major active auxin, and is of highly dynamic nature. The regulation of PAT involves auxin influx transporters of the AUXIN1 (AUX) and LIKE-AUX1 (LAX) types, efflux carrier proteins of the ATP-binding cassette (ABC) and PIN-FORMED (PIN) families, and PINOID family kinases that control the intracellular localization of PINs (Bennett *et al.*, 2014; Geisler *et al.*, 2017).

Studies on petunia (*Petunia hybrida*) cuttings revealed early IAA accumulation in the stem base as dependent on PAT and essential for subsequent AR formation (Ahkami *et al.*, 2013), and highlighted the excision-induced transcriptional fine-tuning of the auxin transport machinery that involved auxin transporters as well as PINOID kinases (Druege *et al.*, 2014). Reviewing these findings in context with other related studies, Druege *et al.* (2016) postulated a model where PAT and cutting off from the basipetal auxin drain are considered as important principles generating early accumulation of IAA in the rooting zone. Further being linked to wound-induced biosynthesis of jasmonic acid (JA) and ethylene (ET), IAA accumulation was suggested to trigger self-regulatory canalization and maximization to responding target cells, there inducing the programme of AR formation.

The important roles of PAT and auxin allocation to particular cells as principles of AR induction and subsequent AR differentiation were highlighted in arabidopsis (*Arabidopsis thaliana*) by tissue-specific monitoring of molecular factors that control auxin homeostasis and by functional analysis of target genes in mutants. In the hypocotyls of de-rooted seedlings, early auxin maxima were identified via *pGH3-2:GUS* in pericycle cells as sites of subsequent AR primordium formation, whereas AR formation was reduced by mutations of *PIN1*, *PIN3*, *PIN7* and *ABCB19* (Sukumar *et al.*, 2013). In isolated TCLs and intact hypocotyls, a local auxin maximum is first initiated in the root founder cells and thereafter directed to the tip of the developing AR meristems (Della Rovere *et al.*, 2013). The *DR5*-reported maximum of auxin perception follows a co-ordinated expression of *LAX3* and of *PIN1*, while the signals are reinforced by exogenous auxin (Della Rovere *et al.*, 2013).

The excision-induced formation of ARs in arabidopsis leaves can be inhibited by a chemical blocker of PAT (Liu *et al.*, 2014) and is associated with early accumulation of IAA and a rise in *DR5* promoter activity, starting in leaf mesophyll cells and then reaching the vasculature near the cutting site (L.Q. Chen *et al.*, 2016; Bustillo-Avendaño *et al.*, 2018). *YUCCA* (*YUC*) genes encoding flavin-containing monoxygenases that convert indole-3-pyruvate to IAA are important controllers of auxin homeostasis in these explants. Using *yuc* mutants and monitoring local *YUC* expression, L.Q. Chen *et al.* (2016) demonstrated that *YUC2* and *YUC6* contribute to the basic auxin level in the leaf, whereas *YUC1* and *YUC4* are excision induced, first in mesophyll cells above the cutting site and thereafter in the procambium and vascular parenchyma, where AR formation starts (*YUC4*). Transgenic or chemical inhibition of *YUC* expression or function, respectively, inhibited AR formation. However, the finding that *YUC1* and *YUC4* are also induced in attached wounded leaves without rooting emphasizes the dependence of AR formation on the disconnection of the leaf from the vascular continuum of the whole plant and on the site of disconnection in relation to the auxin polarity of the leaf. *YUC6* is further highly expressed during early stages of AR primordium formation in entire seedlings and TCLs, localizing in the AR tip (Della Rovere *et al.*, 2013). Excision-induced AR formation in arabidopsis further depends on the function of proteins of the TRYPTOPHAN AMINOTRANSFERASE OF ARABIDOPSIS (TAA) family, which control the auxin biosynthesis upstream of YUC (Sun *et al.*, 2016), and on the auxin-independent *NAM–ATAF1/2–CUC2* (*NAC*) pathway (X.D. Chen *et al.*, 2016).

Under low auxin levels, specific auxin/IAA (Aux/IAA) proteins recruit TOPLESS (TPL) to exert their repressive function on specific AUXIN RESPONSE FACTORS (ARFs), which are transcriptional regulators of auxin-responsive genes. IAA directly binds to the TRANSPORT INHIBITOR RESPONSE 1/AUXIN-SIGNALLING F-BOX (TIR1/AFB) component of the SKP/CULLIN/F-BOX (SCF)–TIR1/AFB complex and to Aux/IAA repressor proteins. This allows the ubiquitination and subsequent proteasomal degradation of Aux/IAA proteins so that the ARFs are released from repression. Aux/IAA proteins further provide cross-nodes to other plant hormones such as CKs, ET, JA and brassinosteroids (reviewed in Druege *et al.*, 2016).

*ARF6* and *ARF8* vs. *ARF17* function as positive vs. negative key regulators of de-etiolation-induced AR formation in intact hypocotyls of arabidopsis (Gutierrez *et al.*, 2012). In *Eucalyptus globulus*, far-red light acclimation of donor plants enhances AR formation in cuttings compared with white light acclimation, which is correlated with *ARF6* and *ARF8* expression levels during AR formation (Ruedell *et al.*, 2015). Despite strong support for the involvement of microRNAs (miRNAs) in hormone-controlled plant development (Curaba *et al.*, 2014), investigations of the role of miRNAs in AR formation remain very limited. During etiolation-induced AR formation in the hypocotyls of intact arabidopsis seedlings, miR160 reduces the transcript levels of *ARF17*, whereas miR167 reduces the expression of *ARF6* and *ARF8* (Gutierrez *et al.*, 2009). Both miRNAs are subject to feedback control by auxin via *ARF6* and *ARF17* expression. In a recent study in apple (*Malus domestica*), overexpression of the double-stranded RNA-binding protein MdDRB1 led to the downregulation of the auxin-associated miRNAs miR160, miR164 and miR393, which was correlated with increased transcript levels of their target genes *MbARF10* and *MbARF16*, *MbNAC1* and *MbTIR1*, respectively, and with enhanced AR formation in microcuttings (You *et al.*, 2014). Interestingly, in arabidopsis, miR160-targeted *ARF10* expression is a positive factor for initiation and formation of callus (Liu *et al.*, 2016) and together with the miR160-targeted *ARF16* expression controls the differentiation of distal columella stem cells and the formation of root cap in the primary root tip, further involving *WUSCHEL-related HOMEOBOX5* (*WOX5*) and *PLETHORA* (*PLT*) as downstream transcription factor (TF) genes (Ding and Friml, 2010).

Considering the phase-specific and auxin-responsive transcriptional regulation of several genes encoding putative Aux/IAA proteins and SMALL AUXIN UP RNA (SAUR) proteins in cuttings of several plant species, Druege *et al.* (2016) suggested specific Aux/IAA–ARF modules as important auxin codes that control the distinct phases of AR formation, and proposed SAUR proteins, that are also linked to ET and JA, to be involved in downstream implementation of the hormone-mediated processes. Degradation of the Aux/IAA proteins is probably facilitated by nitric oxide (NO) which provides a linkage to the frequently observed wound- and auxin-responsive accumulation of NO and hydrogen peroxide (H_2_O_2_) that have promotive influences on AR formation (reviewed in Druege *et al.*, 2016). Recently, in cotyledon segments of *Mangifera indica*, the local upregulation of several *Aux/IAA-like* genes and one *SAUR-like* gene in proximal cut surfaces compared with distal cut surfaces was related to exclusive AR formation at the proximal cut surfaces (Li *et al*., 2016). Interestingly, a recent study of AR formation in leaf explants of arabidopsis mutants highlighted that the Aux/IAA proteins IAA18, IAA14 and IAA28 are required to mediate auxin signalling during vascular proliferation, AR initiation and during both processes, respectively (Bustillo-Avendaño *et al.*, 2018).

A strong transcriptional regulation of auxin-responsive *GRETCHEN HAGEN3* (*GH3*) genes was monitored in the stem base of cuttings of several plant species (reviewed in Druege *et al.*, 2016). *GH3* genes may encode IAA-amidosynthetases conjugating IAA to amino acids, but may also have other functions in AR formation (Gutierrez *et al.*, 2012). Recently, Cano *et al.* (2018) showed that the poor AR formation of a specific carnation cultivar was correlated with higher levels of *DcGH3.1* transcript and of IAA-Asp at the expense of IAA in the stem base of the cutting during AR induction when compared with a good-rooting cultivar. Furthermore, the AR formation in the poor-rooting cultivar could be partially rescued by chemical inhibition of GH3 enzyme activity.

Transcription factors of the WOX family, the GRAS family [such as SCARECROW (SCR) and SHORTROOT (SHR)] and the AINTEGUMENTA-LIKE (AIL) family, belonging to the APETALA 2/ETHYLENE RESPONSE FACTOR (AP2/ERF) domain, exert important control functions during primary root and lateral root (LR) development, linking auxin signalling with cell specification and patterning, in addition to being involved in the feedback regulation of local auxin homeostasis (Ding and Friml, 2010; Horstman *et al.*, 2014). In petunia cuttings, genes of the *AIL* and *GRAS* families, such as *PLT-*, *SHR*- and *SCR*-like TF genes, are upregulated during AR formation (Bombarely *et al.*, 2016). Specific genes show a phase-dependent expression pattern, and *AP2*-like genes include several *ETHYLENE RESPONSE FACTOR* (*ERF*) clades that are induced by wounding in leaves, indicating overlap with the ET signalling pathway. GRAS TFs, which mediate auxin control of cell fate in a phase- and cell-type-dependent manner, are assumed to be important factors in the better rooting capacity of juvenile compared with mature cuttings of woody plants (reviewed in Diaz-Sala, 2014). The function of *GRAS*-, *AP2*- and *WOX*-type TF genes in excision-induced AR formation has been examined in arabidopsis and poplar (*Populus* spp.). In cuttings of *Populus trichocarpa*, the expression of the *AP2/ERF* gene *PtAIL1* is enhanced during the differentiation of AR primordia, while the overexpression or downregulation of *PtAIL1* increases or decreases the extent of AR formation, respectively (Rigal *et al.*, 2012). Expression of the *AP2/ERF* gene *PtaERF003* in *Populus tremula* × *Populus alba* is induced by auxin and was shown to control the intensity of AR formation in cuttings, probably acting as a broad regulator of growth (Trupiano *et al.*, 2013). While null *SHR* and *SCR* mutants show reduced AR formation in arabidopsis TCLs, increased *SCR* expression in wild-type plants starts in the founder cells of ARs and persists in the primordia and elongating ARs (Della Rovere *et al.*, 2015). Indeed, excised leaves of either *shr* or double *plt1 plt2* mutants show reduced AR formation, and triple *shr plt1 plt2* mutants are unable to initiate AR formation, supporting the requirement for the combined activity of SHR, PLT1 and PLT2 in this process (Bustillo-Avendaño *et al.*, 2018).

Expression of *WOX5* characterizes the early derivatives of root founder cells, *in planta* and in *in vitro* cultured TCLs of arabidopsis, and thereafter is co-localized with the auxin maximum in the quiescent centre cells within the AR primordium (Della Rovere *et al.*, 2013). Involving mutation and overexpression of target genes, it was shown that the establishment of root founder cells in arabidopsis leaf explants depends on auxin-mediated, *YUC*-dependent activation of *WOX11* and *WOX12* that control subsequent upregulation of *WOX5* and *WOX7* and of two genes of the *LATERAL ORGAN BOUNDARIES DOMAIN* (*LBD*) family (Liu *et al.*, 2014; L.Q. Chen *et al.*, 2016; Hu and Xu, 2016). AR formation is dependent on *LBD29* expression, and mutations of *WOX5/7* inhibited root primordium formation, suggesting important functions of LBDs and WOX5/7 in the transition of root founder cells to root primordium formation (Liu *et al.*, 2014; Hu and Xu, 2016). Constitutive overexpression of either *PeWOX11a* or *PeWOX11b* strongly accelerates AR formation in *Populus deltoides* × *Populus euramericana* cuttings, increases the number of ARs and further induces ectopic roots in the aerial parts of plants (Xu *et al.*, 2015).

Acting downstream of auxin and GRAS TFs, cyclins (CYCs) and cyclin-dependent kinases (CDKs) are important regulators of the cell cycle and respond to other hormones and to sugars (Komaki and Sugimoto, 2012). Upregulation of *CYCA*, *B* and *D*-type genes has been reported during the induction phase in cuttings of *Pinus contorta*, *Quercus suber*, petunia, carnation and *Vigna radiata* (reviewed in Druege *et al.*, 2016). The functional contribution of *CYCB1.1* to AR regeneration in leaf explants of arabidopsis has recently been shown by mutant analysis (Bustillo-Avendaño *et al.*, 2018).

**Fig. 1.**
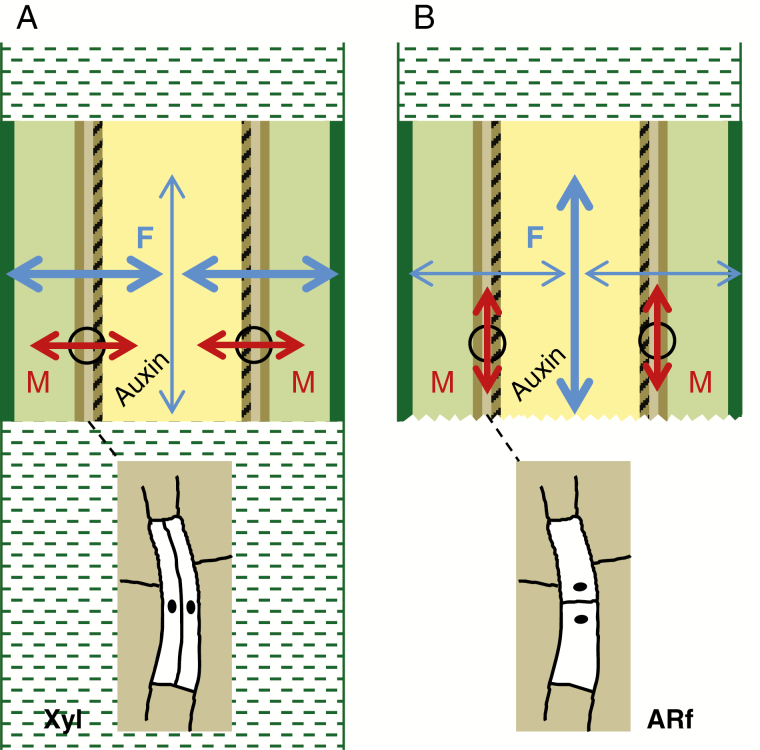
Conceptual model of mechanic effects on microtubule orientation and the resulting orientation of cell division in cuttings. In the stem bases of shoot tips, starting from the outside, the different colours represent the epidermis, cortex, phloem, cambium, xylem and pith tissues. Dashed zones illustrate the apical (A, B) and basal (A) stem connected to the stem bases when the shoot tips are attached to the stock plant (A) and after excision (B). Black circles indicate exemplary positions of cambium cells, while the sketches shown below illustrate their periclinal (A) vs. anticlinal (B) cell division. Blue arrows indicate the direction of mechanical gradients, while the thickness of lines indicates the magnitude. Red arrows indicate the orientation of microtubules in cambium cells. F, mechanical forces; M, microtubules; Xyl, xylogenesis; ARf, AR formation.

Microtubule (MT)-related transcripts encoding tubulins or MT-associated proteins play essential roles in the control of cell division and elongation, while their action involves modification of the cell wall (Landrein and Hamant, 2013). Transcriptome studies of cuttings of *Pinus contorta*, carnation and *Eucalyptus grandis*, and functional analysis in arabidopsis indicated important roles of MT remodelling and cell wall modification during auxin-induced AR initiation (reviewed in Druege *et al.*, 2016). Mutations in the MT-associated protein MOR1 or the MT-severing protein KATANIN reduced auxin-induced AR primordia formation. In this context, observed changes of local *PIN1* expression, auxin perception as well as MT organization and cell wall properties indicated that a fine-tuned cross-talk between MTs, cell wall components and auxin transport is important for the shift from cell division to cell differentiation during AR formation (Abu-Abied *et al.*, 2015). Because MTs are sensitive to mechanical signals, the authors suggested that mechanical perception is important for co-ordinated organ differentiation. Considering the relationship between mechanical stress and MT orientation in plant cells (Landrein and Hamant, 2013), the mechanical sensitivity of MTs may even be involved in the early response of shoot tip cuttings to excision. When AR formation in explants starts from cambium cells, this involves a change of the orientation of division plates (Altamura, 1996). Recent studies on arabidopsis highlighted that AR formation and xylogenesis start from the same cells but are inversely related to each other (Della Rovere *et al.*, 2015). Thus, in the AR-founder cells of the hypocotyl pericycle, anticlinal orientation of the cell division plane is essential for AR formation, whereas periclinal division of the same cells leads to xylogenesis instead of AR formation. There are several indications that the orientation of MTs in a cell follows the mechanical force gradient, while the mechanical stress probably is perceived in the membranes and involves downstream modification of intracellular auxin transport (Landrein and Hamant, 2013). In a normal cylindrical stem, the outer cell layers are under tension whereas the inner tissues are under pressure (Landrein and Hamant, 2013). Thus, potential root founder cells in the stem still attached to the stock plant are exposed to a mechanical force gradient across the stem axis which favours the same directed orientation of MTs (Fig. 1A). Excision of the cutting eliminates the mechanical forces below the wound site so that an axial mechanical gradient is induced in the stem towards the wound, while the release from the basipetal forces may further contribute to reduction of transversal forces (Fig. 1B). Similar to wounding of pea roots, which induced longitudinal orientation of MTs towards the wound site (Landrein and Hamant, 2013), this may induce axial orientation of MTs in the stem (Fig. 1B). This may give rise to anticlinal orientation of the auxin-induced cell division in root founder cells as an early event of AR formation.

Recently, Abu-Abied *et al.* (2018) showed that specific members of the XI myosin family of motor proteins, which are known to be involved in control of cytoplasmic streaming and organelle trafficking, also control polar localization of *PIN1* in the stele cells of primary roots. In root meristematic cells, their transcripts co-localize with the MTs in the mitotic spindle and thereafter with the cell plate. Interestingly, the number of etiolation-induced ARs formed in intact seedlings was strongly enhanced in a triple myosin XI knockout mutant. Even though the data do not provide an explanation of how elimination of myosins contributed to enhanced AR formation (Abu-Abied *et al.*, 2018), these findings suggest important functions of XI myosins in the control of PAT and cell division during AR formation.

In conclusion, the studies on auxin discussed above support the notion that an isolation- and wound-driven change in the auxin homeostasis of a cutting, which is PAT dependent and may include the systemic stimulation of the auxin source capacity particularly in upper plant parts, triggers a self-regulatory process of auxin canalization and maximization towards responding target cells, where auxin perception via the Aux/IAA–ARF machinery induces and drives the programme of AR formation. The findings further strongly suggest the downstream involvement of TFs of the families of GRAS, AP2/ERF (in particular PLT) and WOX (in particular WOX11 and WOX5) and indicate an important role for auxin-mediated *GH3* regulation in adjusting the IAA pool to the different requirements of AR induction and AR differentiation. Correlative studies reveal SAUR proteins as interesting candidates for future research. Even though the functions of some *ARF* genes have been characterized and such investigations of *Aux/IAA* genes have been started in arabidopsis, further functional analysis of candidate genes and of protein interactions is required to unravel the contribution of distinct Aux/IAA–ARF modules to the different phases of AR formation. This also applies to the role of miRNAs. The findings indicate that the miR160–ARF10/16/17 and miR167–ARF6/8 modules may be important molecular factors controlling AR formation. However, the specific interactions and bottlenecks are obviously dependent on the genotype and the particular system, including the AR-generating tissue and the environment. The revealed important functions of MTs for the implementation of the auxin signal, their obvious sensitivity to mechanical stress and their interaction with motor proteins that modify intracellular auxin transport appear to be hot topics to understand which mechanisms drive the cell towards the root fate.

### Roles of ethylene and jasmonic acid

Wounding is one of the key factors triggering ET biosynthesis. An increase in ET emission and upregulation of genes that control ET biosynthesis and responses, particularly *ERF* genes, have been observed in the stem base of cuttings during excision-induced AR formation (reviewed in Druege *et al.*, 2016). Accordingly, the positive function of ET biosynthesis and signalling in AR formation has been demonstrated in diverse plant species, wherein chemical or genetic inhibition of ET biosynthesis or perception reduces AR formation (Clark *et al.*, 1999; Druege *et al.*, 2014; Leatherwood *et al.*, 2016). In *V. radiata* cuttings, early pulse applications of the ET precursor 1-aminocyclopropane-1-carboxylic acid (ACC) or a blocker of ET perception promote or inhibit AR formation, respectively (De Klerk and Hanecakova, 2008). Because the positive effect of the early ACC pulse is dependent on the subsequent auxin supply, the authors suggested that enhanced ET production increases the responsiveness of cells to auxin. However, the decreased or increased numbers of ARs observed in response to late (between 2 and 7 d after excision) pulses of ACC or the ET blocker, respectively, reflect the inhibitory effects of ET during later stages of AR development (De Klerk and Hanecakova, 2008). This finding is in accordance with the inhibitory effects of ET on AR emergence and elongation in cuttings (reviewed in Druege *et al.*, 2016). Extensive cross-talk between auxin and ET exists in both directions at the levels of metabolism, transport and signalling (Muday *et al.*, 2012; Lee *et al.*, 2017). Accordingly, recent data support positive auxin–ET interactions during etiolation-induced AR formation in arabidopsis seedlings (Veloccia *et al.*, 2016) and during excision-induced AR formation in stem cuttings (Villacorta-Martín *et al.*, 2015; Wang *et al.*, 2016; Quan *et al.*, 2017). Interestingly, a reduced ET response in the tomato mutant *Never ripe* enhances auxin transport in hypocotyls but simultaneously reduces de-etiolation-induced AR formation in the same organs, while application of ACC to the wild type had opposite effects (Negi *et al.*, 2010).

Jasmonic acid is another plant hormone that accumulates in plant tissues within a few minutes after physical damage and exerts its physiological activity in a manner dependent on its jasmonoyl-l-isoleucine (JA-Ile) conjugate that interacts with the COI1 (CORONATINE INSENSITIVE1) F-box protein of the SCF^CoI1^ complex as receptor (Wasternack and Song, 2017). JA levels in the basal stem of rooting-competent cuttings from petunia, carnation and pea are highest shortly after cutting excision and decrease to steady-state levels thereafter (Ahkami *et al.*, 2009; Agulló-Antón *et al.*, 2014; Rasmussen *et al.*, 2015). There is further indication from TCLs of arabidopsis that JA accumulation can also be stimulated during AR formation by conversion of the natural IAA precursor indole-3-butyric acid (IBA) to IAA, possibly involving NO-mediated upregulation of JA biosynthetic genes (Fattorini *et al.*, 2017). In petunia, a transgenic reduction of wound-induced JA and JA-Ile levels impairs AR formation (Lischweski *et al.*, 2015). A pulse of low JA administered to pea stem cuttings enhances AR formation in rooting-competent young cuttings (Rasmussen *et al.*, 2015). According to these findings, application of low concentrations of methyl jasmonate (MeJA) to IBA-containing root induction medium of dark-grown tobacco (*Nicotiana tabacum*) or arabidopsis TCLs enhanced AR formation (Fattorini *et al*., 2009, 2017). In tobacco, this response corresponded to enhanced *in situ* expression of marker genes for mitotic activity and higher numbers of meristematic cell clusters, whereas high MeJA concentrations were inhibitory to AR formation (Fattorini *et al.*, 2009).

Continuous supply of JA to the rooting media of intact seedlings of arabidopsis or de-rooted petunia seedlings was found to have no effect at low concentrations but inhibits de-etiolation-induced and excision-induced AR formation, respectively, when applied at higher levels (Gutierrez *et al.*, 2012; Lischweski *et al.*, 2015). Analysis of arabidopsis at the molecular level provided strong support for the concept that de-etiolation-induced AR formation in hypocotyls is subject to auxin–JA cross-talk. Thus, auxin contributes to the downregulation of JA signalling during AR initiation through the interactions of ARF6, ARF8 and ARF17 with GH3.3, GH3.5, GH3.6 and GH3.11 proteins that control the conjugation balance of JA to JA-Ile vs. its physiologically inactive amino acid conjugates (Gutierrez *et al.*, 2012). In rooting-competent cuttings of petunia, carnation and pea, high JA levels in the stem base are followed by IAA maxima (Ahkami *et al*., 2009, 2013; Agulló-Antón *et al.*, 2014; Rasmussen *et al.*, 2015). In contrast, the rise in JA is delayed in low-rooting old pea cuttings that do not show a subsequent IAA peak but instead show a negative rooting response to JA application (Rasmussen *et al.*, 2015). These findings support the idea that the positive effect of JA on AR induction might be concentration dependent and related to subsequent IAA homeostasis. This hypothesis is further supported by a very recent study of Fattorini *et al.* (2018), which was published during the review process of the present article and considered the involvement of jasmonate and ET in AR formation vs. xylogenesis in hypocotyls of intact seedlings and in TCLs of arabidopsis. Application of 0.01 μm MeJA stimulated AR formation in both systems but not in the *coronatine insensitive1-*16 (*coi1-16*) mutant defective in JA signalling. Monitoring of auxin levels in the TCLs revealed that MeJA raised the IAA level when first AR cell clusters were formed, without affecting the expression of *ARF6*, *ARF8* or *ARF17*. Application of 0.1 μm MeJA also promoted AR formation in intact seedlings but failed to induce such a response in TCLs which showed an excision-induced early accumulation of endogenous JA. In contrast, MeJA supplied at micromolar concentrations enhanced xylogenesis in both systems, corresponding to enhanced expression of *ARF17.* Furthermore, the response of AR formation in TCLs to the ET precursor ACC was depressed in mutants defective in JA biosynthesis or signalling, whereas the rooting response to MeJA was depressed in the ET signalling mutant *ein3/eil1*. Further considering that a combination treatment of TCLs with ACC and MeJA indicated antagonistic effects of ET and JA on AR formation, Fattorini *et al.* (2018) postulated a three-way interaction of JA, auxin and ET on the competition between AR formation and xylogenesis.

The application of MeJA to young seedlings or to TCLs of arabidopsis growing in IBA-containing medium increases the expression of *ASA1* (*ANTHTHRANILATE SYNTHASE a1*) (Sun *et al.*, 2009; Fattorini *et al.*, 2017). In seedlings, the *ASA1* response is dependent on COI1, while MeJA further increases the expression of *YUC2*, and the level of IAA in the seedlings stimulates the transcription of *PIN1*, *PIN2* and *AUX1* in roots and alters the endocytosis and plasma membrane accumulation of the PIN2 protein (Sun *et al.*, 2009, 2011). Hentrich *et al.* (2013) showed that the MeJA-induced accumulation of IAA in aerial tissues and roots of arabidopsis is dependent on the function of *YUC8* and *YUC9*, while *YUC9* expression can be stimulated by wounding and is dependent on the COI1 signal transduction pathway. Most interestingly, wound-induced and JA-mediated upregulation of amidohydrolases, some of which are induced shortly after the excision of petunia cuttings (Druege *et al.*, 2014), have recently been shown simultaneously to promote auxin signalling via the release of IAA and attenuate JA signalling via hydrolysis of JA-Ile (T. Zhang *et al.*, 2016).

In conclusion, current knowledge points to the positive effects of ET during the early induction of ARs, while the functional contribution of ET during the later stages of excision-induced AR formation remains unclear. Considering the complex findings on the role of JA, we propose that early, particularly wound-induced, JA accumulation stimulates AR formation in cuttings via IAA accumulation in the stem base and/or canalization towards AR source cells, while the enhanced biosynthesis and de-conjugation of IAA and the modified expression and distribution of auxin transporters are also involved. The accumulated JA may further induce NAC proteins (Nuruzzaman *et al.*, 2013), one of which has an auxin-independent function in excision-induced AR formation in arabidopsis leaf explants, possibly via Cys-endopeptidase-mediated degradation of extension proteins in the cell wall (X.D. Chen *et al.*, 2016). Ahkami *et al.* (2009) discussed the control of apoplastic invertases by JA as found in tomato. Thus, JA may also act via the regulation of sink establishment. At high concentrations, JA may promote xylogenesis rather than AR formation, while the cross-talk between JA and ET may be critical for the competition between both processes. Elucidation of the phase-specific function of auxin–JA–ET cross-talk during AR formation in cuttings is an important topic for future research.

### Interactions with cytokinins and strigolactones

According to the phenomenon that low CK to auxin ratios in the cultivation medium favour root regeneration in explants, the hypothesis of an inhibitory role of high CK levels and a high CK to auxin ratio in AR induction has received broad experimental support. Thus, the CK deficiency and depression of the CK response in arabidopsis mutants enhanced AR formation (Werner *et al.*, 2003; Higuchi *et al.*, 2004), whereas the overexpression of a CK type-B response regulator in *Populus tremula × Populus alba* reduced AR formation in cuttings (Ramirez-Carvajal *et al.*, 2009). Environmentally, developmentally or genetically based increases in CK levels or the CK to auxin ratio in the stem base during the induction phase are correlated with decreased AR formation in cuttings of diverse species (Agulló-Antón *et al.*, 2011; Rasmussen *et al.*, 2015; Villacorta-Martín *et al.*, 2015). These results are in accord with the general view that the lack of root-derived CKs after cutting excision contributes to AR induction. However, CKs may have a promotive influence during the first hours after cutting excision via early stimulation of the cell cycle (da Costa *et al.*, 2013). In hypocotyls of intact arabidopsis seedlings, CKs exhibit an obvious early promotive function of canalization and a maximization of auxin levels during AR primordium development via the restriction of the *LAX3* and *PIN1* expression domains (Della Rovere *et al.*, 2013). Interestingly, a novel study by Bustillo-Avendaño *et al.* (2018) involving several CK mutant combinations shed some light on the signalling cross-talk between auxin and CK during early stages of AR formation in arabidopsis leaf explants. In their working model of AR formation in arabidopsis leaf explants, local CK biosynthesis in the vascular region near the wound in combination with the PAT-induced auxin maximum in this region induces endogenous callus formation. Subsequently, additional reprogramming occurs in a sub-set of these cells, leading to the specification of root founder cells and subsequent AR formation, which is inhibited by CK activity (Bustillo-Avendaño *et al.*, 2018).

Strigolactones (SLs) are a recently characterized group of plant hormones that are derived from the carotenoid biosynthetic pathway and regulate shoot and root development. In arabidopsis, a 9-*cis*/all-*trans*-β-carotene isomerase (encoded by *AtD27*), carotenoid cleavage dioxygenase (CCD) 7 and CCD8 (encoded by *MAX3* and *MAX4*) and a cytochrome P450 (encoded by *MAX1*) are involved in SL biosynthesis which mainly occurs in roots, while the F-box protein MAX2 is important for SL signalling (Al-Babili and Bouwmeester, 2015). In arabidopsis and pea, SLs have been reported to impact AR formation mostly in a negative manner (Rasmussen *et al.*, 2012; Urquhart *et al.*, 2015). In accordance with this role, several genes controlling SL biosynthesis and perception were downregulated in the stem base of petunia cuttings during AR formation (Bombarely *et al.*, 2016). However, the evaluation of mutants of *Oryza sativa* with impaired SL biosynthesis or signalling indicated a positive role for SLs in AR formation (Sun *et al.*, 2015). In arabidopsis, SLs decrease the expression of *CYCB1* and limit AR numbers by inhibiting the first division of founder cells (Rasmussen *et al.*, 2012; Brewer *et al.*, 2013). Considering that SLs can reduce PAT in stems, Rasmussen *et al.* (2012) suggested that SLs may inhibit AR formation via inhibition of PAT, thereby repressing auxin levels in the AR-generating cells. According to this hypothesis, mutations of genes controlling SL biosynthesis or application of synthetic SL enhanced or reduced the shoot–root transport of IAA as well as the expression of several *PIN* genes and *DR5*::*GUS* in the root–shoot junction during AR formation in rice seedlings (Sun *et al.*, 2015). However, auxin may promote SL biosynthesis by enhancing the expression of genes encoding CCD7 and CCD8 (Al-Babili and Bouwmeester, 2015). Thus, cuttings can be expected to be subject to auxin–SL cross-talk in both directions.

Additionally, modifications of SL biosynthesis altered the levels of CKs (Beveridge *et al.*, 1997). Furthermore, SLs may modify the linkage between light and AR formation, and interact with sugar metabolism. In the hypocotyls of arabidopsis, the expression of *MAX3* and *MAX4* is light induced (Rasmussen *et al.*, 2012). Defects in SL biosynthesis in pea completely abolish a positive dark response of AR formation in the lower epicotyl of intact plants (Urquhart *et al.*, 2015). There is evidence that SLs are involved in modulating sugar metabolism and the response to control root development, and vice versa. Arabidopsis mutants defective in expression of *MAX1* or *MAX2* showed a decreased inhibition of root emergence from seeds induced by a high glucose or sucrose supply, and seedlings of both mutants show lower levels of glucose and fructose compared with wild-type plants (Li *et al.*, 2016). Additionally, investigations on *Rosa hybrida* showed that sugars such as sucrose can downregulate genes controlling SL transduction (Barbier *et al.*, 2015).

In conclusion, the findings on the role of CKs increasingly indicate an early positive function during cell dedifferentiation, an antagonistic function to auxin during the determination of root founder cells and a co-operative interaction with auxin during subsequent AR differentiation. The findings on the role of SLs indicate the possible existence of sugar–SL–auxin cross-talk, which should be considered in future studies on AR formation.

## RELATIONSHIP BETWEEN WOUND SIGNALLING, EPIGENETIC MODIFICATIONS AND PLANT HORMONE ACTION

Considering the diverse cellular origins of excision-induced ARs (Altamura, 1996), the initial cell reprogramming towards root competence or immediate fate conversion of root-competent cells to AR founder cells may initiate the process in cuttings (da Costa *et al.*, 2013; Bustillo-Avendaño *et al.*, 2018). Studies of arabidopsis indicated that wound-induced AP2/ERF TFs of the WIND (WOUND INDUCED DEDIFFERENTIATION) family activate the local CK response at the wound site, which in turn promotes cell de-differentiation during wound healing (Iwase *et al.*, 2011). Expression of *AtWIND1* induces ectopic callus formation in *Brassica napus*, tomato and *N. tabacum* (Ikeuchi *et al.*, 2013). In the petioles of arabidopsis leaf explants, *WIND1* is expressed in proliferating vascular cells near the excision site, but is downregulated thereafter in the new root primordia (Bustillo-Avendaño *et al.*, 2018).

During normal plant development, many central regulators of regeneration are epigenetically silenced to prevent inappropriate cellular reprogramming, mainly through histone hypoacetylation, DNA methylation and chromatin remodelling (Ikeuchi *et al.*, 2016; Yamamuro *et al.*, 2016). Findings regarding other developmental processes indicate important links between wound signalling, epigenetic control machinery and phytohormones. Avivi *et al.* (2004) showed that the acquisition of pluripotency in arabidopsis leaf protoplasts is associated with reduced DNA methylation of specific chromosomal domains and corresponding upregulation of several members of the *NAC* gene family of TFs, some of which are also involved in excision-induced AR formation in arabidopsis leaf explants (X.D. Chen *et al.*, 2016). Recent evidence has emphasized the role of plant hormones, particularly auxin, in chromatin opening involving DNA methylation and demethylation (Yamamuro *et al.*, 2016). In arabidopsis, the Polycomb repressive complex (PRC)1/2 was found to act as important gene repressor via the trimethylation of Lys27 of histone H3 (H3K27me3) (Pulianmackal *et al.*, 2014). Interestingly, members of the *WIND* gene family are targets of the PRC machinery, as *PRC2* mutation enhances *WIND3* expression (Ikeuchi *et al.*, 2016). DNA methylation can also affect auxin homeostasis and signalling. Analysis of gene expression in the shoot apical meristem and differentiated leaf cells of *PRC* complex mutants revealed several H3K27me3 target genes (Lafos *et al.*, 2011). Many of them, such as *YUC1*, *YUC4*, *PIN1*, *AUX1*, *LAX3* and *IAA19*, control auxin biosynthesis, transport, perception and signalling, and are involved in AR formation as described before. Interestingly, most ARFs were not direct targets of PRC repression but were controlled by the H3K27me3-mediated modulation of their regulatory miRNAs, including those that interact with ARF6/8 and ARF17 (Lafos *et al.*, 2011), which control AR formation (Gutierrez *et al.*, 2012). Analysis of gene expression and H3K27me3 at corresponding loci during callus formation in arabidopsis leaf explants revealed early histone hypomethylation and increased expression of genes controlling the auxin pathway, including several *GH3* genes (He *et al.*, 2012). The arabidopsis long intergenic non-coding RNA APOLO is transcribed in response to auxin and regulates the PRC-dependent opening of a chromatin loop encompassing the promoter of its neighbouring gene *PINOID* (Ariel *et al.*, 2014), which controls the intracellular localization of PIN transporters.

A decline in AR formation of cuttings along with the maturation of the cutting source tissue has been frequently observed, particularly in woody species (reviewed in Diaz-Sala, 2014). Diaz-Sala (2014) proposed that epigenetic modifications also contribute to such maturation effects. Interestingly, in arabidopsis, both overexpression of miR156 and chemical inhibition of DNA methylation could partially rescue the depressed AR formation in mature explants compared with explants from younger tissues (Massoumi *et al.*, 2017). These results suggest methylation-mediated miRNA activities as important factors controlling maturation effects on AR formation in cuttings (Massoumi *et al.*, 2017).

Dynamic histone deacetylation and acetylation have been proposed to control auxin signalling (Yamamuro *et al.*, 2016). Loss of function of the histone acetylase gene *GCN5* in arabidopsis severely reduces the expression of auxin-inducible *PLT* genes and *CYCB1*, and causes stem cell malfunction in primary roots (Kornet and Scheres, 2009). Based on the interactions between IAA12/BODENLOS (BDL), TPL, HDA19 and GCN5, Yamamuro *et al.* (2016) suggested a model in which the co-repressor TPL recruits the histone deacetylase HDA19 to the IAA12/BDL promoter during ‘auxin-off’ conditions, whereas during ‘auxin-on’ conditions, the histone acetylase GCN5 plays an opposing role and is recruited to auxin-responsive promoters to activate their expression. Interestingly, *HDA6* and *HDA19* are induced by ET and JA (Zhou *et al.*, 2005), while *HDA6* acts as a repressor of ET and JA signalling (Zhu *et al.*, 2011). Specific chromatin remodelling factors determining chromatin structure have also been shown to modulate the auxin distribution by controlling the expression of several *PIN* genes and the responses to plant hormones including auxin, ET and CKs (Yamamuro *et al.*, 2016). For example, the chromatin remodelling factor PICKLE seems to control auxin- and ARF-dependent cell fate specification and cell cycle progression during LR formation through changes in histone modifications while integrating different external and internal signals (Fukaki *et al.*, 2006).

The findings discussed above strongly support the hypothesis that expression of WIND1 is not a crucial factor for excision-induced rhizogenesis *per se* but it is an important prerequisite for AR formation that requires previous de-differentiation of non-rooting-competent AR source cells so that they can be subsequently committed to become a root. In this context, *WIND* and also other molecular factors that control hormone homeostasis and AR formation are subject to the DNA and chromatin structure. These epigenetic factors, either present or modified by early wound- or isolation-induced changes of hormones such as ET or JA, may determine the competence of the involved cells for de-differentiation and/or for their fate conversion to AR founder cells. The functional study of epigenetic factors in relation to the different phases of AR appears to be an important research topic.

## INTEGRATED MOLECULAR REGULATION OF AR FORMATION IN THE STEM BASE

**Fig. 2.**
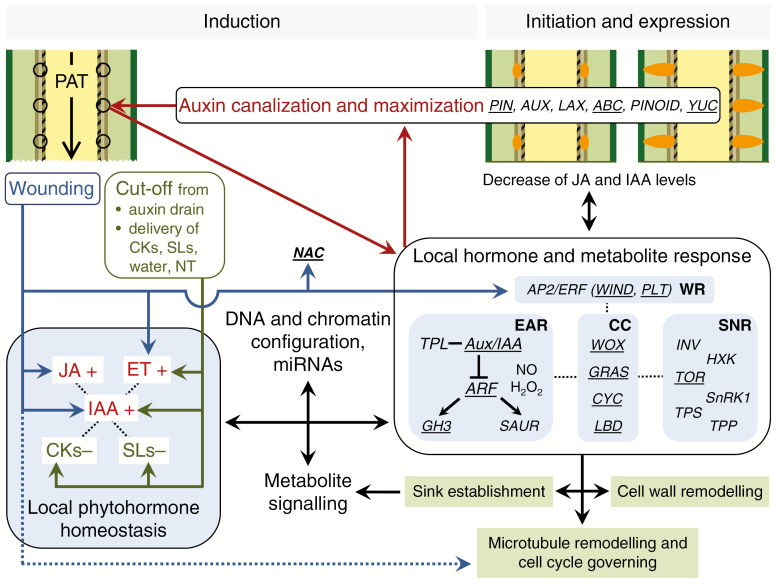
Model of molecular regulation of excision-induced AR formation in the stem base of cuttings. In the stem base, starting from the outside, the different colours represent the epidermis, cortex, phloem, cambium, xylem and pith tissues. Black circles indicate cells where AR formation starts in the cambium as an example. Elliptic and dome-shaped structures in ochre colour indicate clusters of new meristematic cells and developing AR primordia, respectively. Arrows in different colours show the direction of actions. Blue arrows indicate specific effects of wounding, while the broken line represents mechanical effects as illustrated in Fig. 1. Green arrows indicate effects of cutting isolation. Red arrows indicate a self-organizing auxin loop. Plus vs. minus signs indicate increase vs. decrease of hormone concentrations, respectively. WR, EAR, CC and SNR indicate groups of genes controlling the wound response, early auxin response, cell cycle and sugar/nutrient response, respectively. Underlined characters mark those genes whose function in AR formation has been confirmed by mutation or overexpression. ABC, ATP-binding cassette; AP2/ERF, APETALA 2/ETHYLENE RESPONSE FACTOR; ARF, AUXIN RESPONSE FACTOR; AUX, AUXIN1; Aux/IAA, AUXIN/INDOLE-3-ACETIC ACID; CKs, cytokinins; CYC, cyclins; ET, ethylene; GH3, GRETCHEN HAGEN3; GRAS, named after GIBBERELLIC ACID INSENSITIVE, REPRESSOR OF GIBBERELLIC ACID INSENSITIVE and SCARECROW; HXK, hexokinase; IAA, indole-3-acetic acid; INV, invertases; JA, jasmonic acid; LAX, like AUX; LBD, LATERAL ORGAN BOUNDARIES DOMAIN; NAC, NAM–ATAF1/2–CUC2; NT, nutrients; PAT, polar auxin transport; PIN, PIN-FORMED; PINOID, PIN-targeting serine threonine protein kinase; PLT, PLETHORA; SAUR, SMALL AUXIN UP RNA; SLs, strigolactones; SnRK1, sucrose non-fermenting 1-related protein kinase 1; TOR, target of rapamycin; TPL, TOPLESS; TPP, trehalose-6-phosphate phosphatase; TPS, trehalose-6-phosphate synthase; WIND, WOUND INDUCED DEDIFFERENTIATION; WOX, WUSCHEL-related HOMEOBOX. Further explanations are provided in the text.

Based on the literature discussed above, a model regarding the molecular regulation of excision-induced AR formation in the cutting base is illustrated in Fig. 2. Wounding at the cutting surface stimulates the rapid biosynthesis of JA and ET, contributes to an early accumulation of IAA via enhanced biosynthesis and release from conjugation, and enhances the expression of *NAC* and specific *AP2/ERF* TF genes, such as *WIND* and *PLT*. Isolation from the root system reduces the amount of root-sourced CKs and SLs, and the delivery of water and nutrients, which further stimulates ET biosynthesis and simultaneously restricts the rootward transport of auxin, leading to a dramatic rise in IAA during the induction phase. In addition to the induction of wound healing at the cut surface, the plant hormone response initiates autonomous regulation of components of the auxin transport machinery, contributing to the canalization and maximization of auxin, targeting specific reprogrammable cells, most frequently in the cambium or vascular tissues. These respond to changes in hormone homeostasis in a manner determined by their specific DNA and chromatin configurations and the action of miRNAs. Cross-talk with other hormones, sugars and other metabolites (see below) controls the expression of the different TFs and enzymes discussed above that guide the establishment of the new sink in the rooting zone (further explanation below) and the determination, initiation and further differentiation of ARs, involving a mediation of the cell cycle and remodelling of microtubules and the cell wall as major processes. As apparent from Fig. 2, the functions of several molecular factors such as SAUR genes need further investigation.

## NUTRITIONAL AND METABOLIC CONTROL AT THE WHOLE-CUTTING LEVEL

### Mineral status of cuttings, nutrient mobilization and external nutrient application

A deficiency of any essential element, such as carbon or macro- or micronutrients, may limit AR formation, either at a systemic level, by interfering with major physiological processes, or by local effects on processes associated with the induction, initiation and expression of ARs. The separation of a cutting from the existing root system disturbs the influx of nutrients and fixes the amounts of available mineral elements until the nutrient uptake capacity is restored. The important role of the initial endogenous pool of nutrients in the cutting, as determined by the nutritional status of stock plants, has been highlighted in numerous reports on AR formation in the leafy cuttings of diverse species in nutrient-free substrates (Druege *et al*., 2000, 2004; Ahkami *et al.*, 2009; Zerche and Druege, 2009; Agulló-Antón *et al.*, 2011). There is indication from several plant species that AR formation in cuttings can be impaired by low supply of K or Fe to the stock plants (Henry *et al.*, 1992; Tsipouridis *et al.*, 2006). More studies have focused on the role of N in stock plants, which is further linked to carbohydrate metabolism. Studies examining pelargonium (*Pelargonium × hortorum*), *Chrysanthemum indicum*, *Euphorbia pulcherrima* and petunia have shown that the number and length of ARs are positively correlated with the initial total N concentration in the cuttings; in petunia, in particular, this phenomenon is associated with enhanced differentiation of new meristematic cells into fully developed ARs (Druege *et al*., 2000, 2004; Zerche and Druege, 2009; Zerche *et al.*, 2016). Monitoring of K, micronutrients and amino acids as mobile N compounds in the stem base of cuttings during AR formation indicated that AR formation is not only dependent on the initial content of nutrients in the tissues at cutting excision but particularly involves subsequent mobilization and retranslocation within the cutting (Svenson and Davies, 1995; Ahkami *et al.*, 2009). In this context, a higher N supply to petunia stock plants enhanced N allocation to mobile N pools and the amino acid content in leaves, which constitute important source organs for N remobilization, e.g. via glutamine and asparagine, towards the new developing ARs (Zerche *et al.*, 2016). Dong *et al.* (2004) demonstrated that approx. 30 % of the mobilized N in cuttings of *P. trichocarpa* × *P. deltoides* is allocated towards newly formed ARs. Recently, Zerche *et al.* (2016) showed that dark exposure of cuttings, which promotes AR formation in petunia (Klopotek *et al.*, 2010), is another factor enhancing the levels of soluble amino-N, amide-N and amino acids in cuttings, with leaves as important source organs. Considering these findings and the corresponding decreases in carbohydrates and insoluble protein-N, the authors postulated that dark-induced and carbohydrate depletion-mediated proteolysis leads to the mobilization and retranslocation of amino acids in cuttings, with asparagine as an important phloem-mobile component (Zerche *et al.*, 2016). Fewer mobile nutrients are also allocated towards the cutting base. In poinsettia, Fe, Cu and Mo accumulate in the cutting base during early root initiation, followed by additional increases in the concentrations of Mn, B and Zn during primordium elongation and root emergence (Svenson and Davies, 1995). In petunia, Fe, Cu and Zn begin to accumulate in the cutting base as early as 1 d after excision (Hilo *et al.*, 2017).

Several studies of intact plants have revealed complex linkages of N nutrition with homeostasis and signalling pathways of auxin (Vidal *et al.*, 2010; Jin *et al.*, 2012) and CKs (Sakakibara *et al.*, 2006; Kamada-Nobusada *et al.*, 2013). The additional influences of wounding and isolation in excised shoots complicate a clear hypothesis concerning how auxin or CK action might contribute to the effects of N on AR formation in cuttings. Nevertheless, considering arginine as a putative NO donor in plants, Zerche *et al.* (2016) suggested arginine-derived NO signalling as a candidate signal involved in the promotion of AR formation in response to a higher N nutrition of stock plants and dark-induced N mobilization.

Despite the established importance of the mineral composition of the rooting medium based on empirical trials examining the *in vitro* propagation of various plant species, little is known concerning the mechanisms of nutrient uptake in the stem base of cuttings before the emergence of ARs. Transcriptome analysis of the petunia cutting base revealed upregulation of 18 genes involved in the acquisition of N, P, K, S, Fe and Zn during early initiation of the AR primordia (Ahkami *et al.*, 2014). These results suggest that the cutting base acquires the capacity for specific uptake of nutrients, which is characteristic of the root system, and that AR formation may be improved by the local application of certain limiting nutrients to the rooting zone. The general importance of local nutrient effects was shown in petunia cuttings, where local application of combined N–P–K fertilizer to the stem base but not to the leaves during AR emergence improved rooting (Santos *et al.*, 2009).

Table 1 summarizes the reported effects of local nutrient application to the stem base on AR formation in cuttings. These studies, which indicate positive effects of N, K, Ca, B, Fe and Zn, involved various plant species and are often conducted in different growth systems, further affecting the chemical conditions of the rooting zone. In this context, effects of NH_4_^+^ and nitrate (Table 1) might also involve the well-known physiological acidification and alkalinization of the rhizosphere, respectively. Schwambach *et al.* (2005) discussed the extremely low pH reaching 2.6 as a possible factor mediating the inhibitory effect of NH_4_^+^, and considered reversal effects or signalling functions of nitrate similar to those on LR formation as possible mechanisms underlying respective promotive functions on *in vitro* AR formation in *E. globulus.* Recently, Hilo *et al.* (2017) found a promotive influence of NH_4_^+^ on AR formation in petunia cuttings in a pH-buffered hydroponic system. They proposed local acidification of the apoplast of the stem base as a candidate mechanism, which might further involve increased mobilization of Fe (for further discussion see below). Although P deficiency can regulate the root system architecture in intact plants by increasing the number of ARs (Miller *et al.*, 2003), there is no indication in the literature of a similar role for P in cuttings.

**Table 1.** Summary of the reported effects of external nutrient application on AR formation in stem cuttings

Mineral element	Supplied form	Plant species	Reported effect	Reference
N	NaNO_3_ NH_4_Cl	*Eucalyptus globulus* Labill. *Eucalyptus globulus* Labill.	Increased rooting percentage, AR number and length Decreased AR number and length	Schwambach *et al*. (2005, 2015) Schwambach *et al.* (2005)
	Glutamic acid	*Eucalyptus globulus* Labill.	Increased AR number and decreased AR length	Schwambach et. al. (2005)
	(NH_4_)_2_SO_4_	*Petunia hybrida* Vilm.	Increased AR initiation and enhanced meristematic cell division	Hilo *et al.* (2017)
	Arginine Ornithine Glutamic acid	*Malus domestica* L.	Increased rooting percentage and AR number	Orlikowska (1992)
K	KCl	*Cucumis sativus* L. *Phaseolus radiatus* L. *P. vulgaris* L.	Increased AR number	Zhao *et al.* (1991)
Ca	CaCl_2_	*Pisum sativum* L.	Increased AR elongation	Eliasson (1978)
B	H_3_BO_3_ + IBA	*Phaseolus aureus* Roxb.	Increased AR number and length	Middleton *et al.* (1978)
	H_3_BO_3_	*Helianthus annuus* L.	Increased initiation of ARs and meristematic activity	Josten and Kutchera (1999)
Fe	FeEDDHA	*Prunus amygdalus×P. persica*	Increased rooting percentage, AR number and length	Molassiotis *et al.* (2003)
	FeEDDHA	*Petunia hybrida* Vilm.	Increased AR initiation and enhanced meristematic cell division	Hilo *et al.* (2017)
Zn	ZnSO_4_ + NAA	*Mangifera indica* L.	Increased rooting percentage, AR number and total AR length	Yamashita *et al.* (2006)
	Zinc ammonium acetate	*Physocarpus opulifolius* (L.) Maxim.	Increased rooting percentage, rooting quality and stem elongation	Pacholczak and Szydło (2008)

The reported positive effects of local Ca application (Table 1) correspond to its low mobility in the phloem. The percentage of arabidopsis TCLs rooted *in vitro* and the number of ARs were dependent on the optimum Ca dosage (Falasca *et al.*, 2004). These effects were observed when Ca was applied either during AR induction, provided by high IBA and low CK concentrations, or during later stages of AR formation. This finding highlights different Ca functions during AR formation. Both intracellular and extracellular Ca pools are crucial for AR formation, as the application of chelators that scavenge either apoplastic or intracellular Ca strongly reduces rooting parameters in cuttings of *Cucumis sativus* (Lanteri *et al.*, 2006). While extracellular Ca might be associated with cell wall fortification, intracellular Ca acts as a secondary messenger involved in the signal transduction of known triggers of AR formation, such as auxins and NO (Lanteri *et al.*, 2006). Similar to Ca, B is known to be essential for the maintenance of the cell wall structure and is therefore required for cell expansion and division. The reported positive effects of B application on meristematic activity and initiation, and the final number and length of ARs in cuttings (Table 1) can be explained by this function. For decades, the importance of Zn in AR formation has been attributed to its well-known role in the synthesis of tryptophan, which is a precursor of the major auxin form IAA. However, while the promotive effect of ammonium zinc acetate on the rooting of *Physocarpus opulifolius* may be related to this role, the positive effect of Zn on cuttings of *Mangifera indica* in combination with the synthetic auxin 1-naphthaleneacetic acid (NAA) may indicate functions independent on auxin (Table 1).

Fe is a constituent of many enzymes involved in various physiological processes, such as photosynthesis, primary and secondary metabolism, the antioxidant system, DNA replication and gene expression. Furthermore, local availability of Fe has been shown to affect LR elongation via induction of the auxin transporter gene *AUX1*, thus influencing the balance of this hormone (Giehl *et al.*, 2012). In petunia cuttings, Fe was discovered as the most limiting element during AR formation, and a single application of Fe to the stem base dramatically increased the number of ARs, whereas foliar application had no effect (Hilo *et al.*, 2017). Interestingly, basal Fe application affected neither the hormonal balance nor primary metabolism in the cutting base. However, histochemical localization revealed an increased accumulation of Fe as dot-like structures in the nuclei of dividing cambial cells during AR initiation and later in the meristematic cells of ARs. Such specific allocation of Fe and increased transcript levels of mitotic cyclins suggested a role in the division of meristematic cells, possibly by activating ribosome biogenesis (Hilo *et al.*, 2017), which is further discussed and illustrated below in relation to metabolic regulation.

Overall, the available studies do not sufficiently explain the processes underlying the effects of the nutritional status of whole cuttings on adventitious rooting, and the allocation and specialized local functions of nutrients during the distinct rooting phases are mostly unknown. Therefore, mineral nutrition is one of the most unexplored subjects related to the topic of AR formation and requires further thorough investigation.

### Carbohydrate source and sink relationships

Studies on cuttings of ornamental plant species, which are usually subjected to dark incubation after harvest before being planted, have highlighted the importance of the leaf carbohydrate source capacity for intensive AR formation in the stem base. Because respiration and other metabolic processes are not balanced by photosynthesis, dark storage of cuttings causes a decrease in carbohydrate levels. This carbohydrate depletion is positively correlated with temperature and duration of storage, while it is more pronounced in the leaves than in the stem base and usually starts with starch, followed by sucrose (Druege *et al*., 2000, 2004; Rapaka *et al.*, 2005; Klopotek *et al.*, 2010). Due to the well-known positive relationship between leaf N concentrations and the light saturation rate of photosynthesis, higher N levels in cuttings may contribute to increased leaf photosynthesis, enhancing the carbohydrate source capacity. However, N-deficient cuttings show higher starch levels upon excision and maintain higher sugar levels during dark storage than those in N-rich cuttings (Druege *et al*., 2000, 2004; Zerche and Druege, 2009). These findings can be explained by the linkage between nitrogen and carbohydrate metabolism, where carbohydrate biosynthesis and N assimilation into amino acids compete for reduced carbon and energy inputs (Druege *et al.*, 2000). Studies investigating *Chrysanthemum indicum* cuttings indicated that AR formation under high-light conditions might not necessarily be impaired by dark storage-induced carbohydrate losses, but may instead depend on carbon partitioning between sucrose and starch in source leaves (Druege *et al.*, 2000).

Adventitious root formation in the stem base of pelargonium is limited by carbohydrate shortages in leaves when dark-stored cuttings depleted of their carbohydrate reserves experience low-light conditions during subsequent cultivation (Druege *et al.*, 2004). However, a higher light level after planting of the cuttings abolishes the inhibitory effect of a leaf carbohydrate shortage at the time of planting, while the number of ARs is generally correlated with the mean leaf sucrose level during the first week of cultivation (Rapaka *et al.*, 2005). Accordingly, lowering the air temperature during cutting cultivation under low light, which enhances net photosynthesis and increases the levels of sugars (mainly sucrose) in the tissues of cuttings, represses leaf senescence and contributes to improved root formation in the stem base (Druege and Kadner, 2008). Furthermore, a multivariate analysis of parameters determining AR formation in cuttings of *E. pulcherrima* revealed that the positive effect of the leaf sucrose level at the time of planting on AR number and length is less important in the presence of a higher daily light integral (DLI) during the rooting period (Zerche and Druege, 2009). Several studies have confirmed the important contribution of a sufficiently high light intensity to AR formation (Lopez and Runkle, 2008) and the positive relationships between light intensity or DLI, carbohydrate levels in the stem base during rooting and the final intensity of AR formation (Currey and Lopez, 2015; Tombesi *et al.*, 2015). In conclusion, a high and steady export of carbohydrates from source leaves, which is the function of initial carbohydrate reserves and current net photosynthesis, is an important requirement for a high intensity of AR formation in the stem base of leafy cuttings.

Considering earlier conflicting results concerning the light response of cuttings, Klopotek *et al.* (2012) and Tombesi *et al.* (2015) emphasized the importance of maintaining an optimum vapour pressure deficit by adjusting the light intensity and water supply during rooting. Studies on pelargonium and petunia cuttings further revealed that photosynthesis during cutting cultivation is dependent not only on the current CO_2_ supply, the previous light acclimation of the photosynthetic apparatus and current light levels (Rapaka *et al.*, 2005; Klopotek *et al.*, 2012), but also on plant genotype. Hence, pelargonium cuttings show only weak photosynthetic activity and maintain low carbohydrate levels at low light levels (Druege and Kadner, 2008), whereas cuttings of petunia respond to similar environmental conditions with higher net photosynthesis, leading to a quick recovery from dark-induced carbohydrate shortages (Klopotek *et al*., 2010, 2012).

The first detailed biochemical analysis of carbohydrate metabolism in relation to the anatomical stages of the stem base of petunia cuttings was undertaken by Ahkami *et al.* (2009), who highlighted the dynamics on the sink side of the carbohydrate network. These authors demonstrated that AR formation is associated with local changes in carbohydrate-related enzyme activities and a rearrangement of metabolic pathways. Based on the results, three metabolic phases were defined, starting with a sink establishment phase, characterized by apoplastic unloading of sucrose and its cleavage into hexoses, glucose and fructose by apoplastic invertase, followed by the transport of hexose into the cytosol by monosaccharide transporters, where hexoses are utilized for the production of energy necessary for wound healing and cell division, leading to a transient depletion of sugars. The second, recovery phase is characterized by the replenishment of resources and lasts up to 3 d, ending with the formation of new cell clusters. Finally, re-establishment of cell connections in the maintenance phase allows the symplastic transport of sugars from source leaves towards the developing AR primordia in the stem base. The delivered carbohydrates are either used immediately in catabolic processes or transiently stored as starch in the surrounding cortical cells. There, it may serve as an intermediate carbohydrate depot close to the AR-forming cells and may also have developmental functions, e.g. in root cap definition (Altamura *et al.*, 1991; Ahkami *et al.*, 2009). The prominent function of carbohydrates has also been shown in other ornamental plants such as carnation, in which low levels of sucrose in the base of the stem were detected (Agulló-Antón *et al.*, 2011). Here, the initially high glucose level in the stem base was found to be followed by a transient decrease during rooting, reflecting a high energy requirement during rooting that is not initially covered by the carbohydrate influx from the photosynthesizing leaves.

There is increasing support in the literature for the contribution of modified auxin homeostasis in the stem base of cuttings to sink establishment via local stimulation of sucrolytic activity. Inhibition of PAT in petunia cuttings, which eliminates the auxin peak, reduces the activities of apoplastic invertase and vacuolar invertase in the stem base (Ahkami *et al.*, 2013). The authors responsible for these findings postulated that PAT and the resulting early auxin accumulation favour the accumulation of sucrose and the co-transportation of amino acids in response to the enhanced invertase-dependent sink activity. In accord with this view, pulse treatments of cuttings with auxin stimulated the activities of vacuolar, cytosolic and apoplastic invertase and sucrose synthase in the stem base of carnation (Agulló-Antón *et al.*, 2014). Furthermore, auxin pulses advanced the peak of soluble proteins in the phloem tissue of the stem base of *Malus hupehensis* (Zhang *et al.*, 2017) and altered the expression of several genes that control amino acid transport, biosynthesis and metabolism, in a phase- and gene-specific manner in cuttings of *Robinia pseudoacacia* (Quan *et al.*, 2017).

The contribution of carbon allocation and invertases to the stimulation of AR formation in response to dark pre-exposure of petunia cuttings has been investigated in the stem base and the shoot apex as competing sinks. During dark pre-exposure, higher activities of cytosolic and vacuolar invertases are maintained in both sinks compared with cuttings growing under light (Klopotek *et al.*, 2016). The activity of apoplastic invertase increases specifically in the stem base under both light and dark conditions, coinciding with increased expression of the corresponding gene. The presented results indicate that dark exposure before planting enhances the carbon sink competitiveness of the rooting zone against the upper shoot and that the expression and activity of invertases contribute to the shift in carbon allocation towards the developing ARs after exposure of the cuttings to light (Klopotek *et al.*, 2016).

There is an indication from the literature that auxin is also involved in dark-stimulated and light-spectrum-mediated AR formation involving carbohydrate allocation and metabolism. Dark storage of carnation cuttings increases auxin levels, the auxin/CK ratio and AR formation in the stem base of carnation cuttings compared with storage under low light (Agulló-Antón *et al.*, 2011). The application of auxin to cuttings before planting stimulates the subsequent accumulation of sugars in the stem base of the non-stored cuttings, but has a less pronounced effect on the previously dark-stored cuttings. Treatment of *E. globulus* stock plants with far-red light, which enhances AR formation in excised cuttings, not only stimulates the expression of three genes putatively controlling auxin biosynthesis (*YUC3*) and auxin efflux (*PIN1* and *PIN2*) in cuttings during the induction phase, but further induces the expression of *SUS1* and *SUC5* putatively encoding a sucrose synthase and sucrose transporter, respectively, during the later stage of AR formation (Ruedell *et al.*, 2015).

**Fig. 3.**
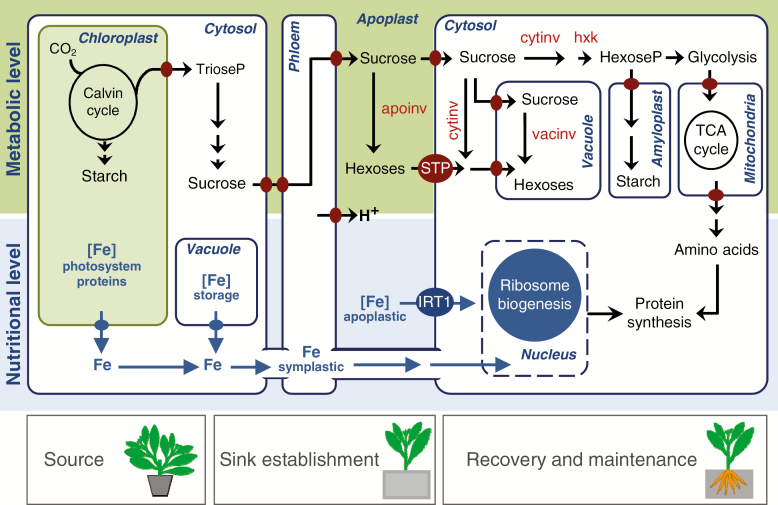
Model of processes controlling the metabolic and Fe-mediated regulation of AR formation in petunia cuttings. Crucial metabolic pathways, enzymes and metabolites are assigned to the involved compartments at the source and sink sites. Arrows indicate directions of transport or conversion of iron in blue colour. Red and blue discs indicate metabolite and iron transporters, respectively. apoinv, apoplastic invertase; cytinv, cytosolic invertase; hxk, hexokinase; IRT1, iron-regulated transporter 1; STP, monosaccharide transporter; vacinv, vacuolar invertase. Further explanations are provided in the text.

Based on the findings summarized above, a linkage between the metabolic regulation and Fe limitation of adventitious rooting in petunia (Hilo *et al.*, 2017; Table 1) is postulated as illustrated in Fig. 3, where the protein synthesis in the rooting zone is considered as the nodal point between both factors. The sugar supply is a critical metabolic bottleneck essential for the energy production and metabolic activity generating distinct sugar and amino acid profiles, which may specifically control different processes of AR formation, as further emphasized by Agulló-Antón *et al.* (2014). Among the critical mineral elements, Fe plays a crucial role in ribosome biogenesis, which becomes a bottleneck for the active cell division and differentiation of meristematic cells. Isolation of cuttings from the stock plant interrupts the transport of assimilates and mineral elements which, together with JA and auxin signals, leads to the establishment of a new sink in the cutting base. At this stage, the key assimilates and Fe stored by the stock plant are locally mobilized to supply the AR source cells. Local acidification of the apoplast stimulates the activity of apoplastic invertases and facilitates the mobilization of Fe precipitated in the apoplast. With the progression of AR formation, the recovery of long-distance sugar transport stimulates biosynthetic processes and energy production in the developing AR meristems that will form the body of ARs. Activation of IRT (iron-regulated transporter)-type transporters at this stage allows immediate uptake of Fe from the rooting medium.

### Metabolite signalling and cross-talk with plant hormones

Although the promoting effect of sugars on AR formation has been supported by several *in vitro* studies, these studies did not provide a coherent picture of the particular contribution of sugars to specific phases of AR formation (Takahashi *et al.*, 2003; Correa *et al.*, 2005; Yasodha *et al.*, 2008). Far beyond providing energy, carbon and osmotic activity, sugars act as signals and mediate the development, growth and stress responses of plants by modulating gene expression (Rolland *et al.*, 2006; Smeekens *et al.*, 2010; Eveland and Jackson, 2012).

Thus far, there has been only an indirect indication that the effects of sugars on AR formation in cuttings might involve signalling functions. Recently, the conserved glucose-sensing hexokinase (HXK) pathway, trehalose-6-phosphate (T6P), sucrose-non-fermenting-1-related protein kinase-1 (SnRK1) and the target of the rapamycin (TOR) kinase pathway have been identified as important interlinked regulatory components mediating the effects of C nutrient status on plant growth and development (Rolland *et al.*, 2006; Smeekens *et al.*, 2010). Interestingly, in the stem base of petunia cuttings, several genes encoding HXK, one SnRK1 gene homologue and two genes encoding a trehalose-6-phosphate synthase (TPS) and a trehalose-6-phosphate phosphatase (TPP) were upregulated during the induction phase, when the sugars are transiently depleted and the new carbohydrate sink is established (Ahkami *et al.*, 2014). Generally, SnRK1 is enhanced in activity with sugar depletion, can mediate cell cycle progression and affects phase transitions during plant development while interacting with CDKs (Baena-Gonzalez, 2010; Smeekens *et al.*, 2010). TPS and TPP catalyse the synthesis of T6P downstream of glucose and its de-phosphorylation, respectively, while T6P inhibits SnRK1 activity (Eastmond *et al.*, 2003; Smeekens *et al.*, 2010). Specific members of the TPS gene family, which are apparently catalytically inactive, showed sugar-dependent expression patterns, while the AtTPS1 protein interacted with the cell cycle-dependent kinase CDKA1 and kinesin KCA1 (Smeekens *et al.*, 2010). Considering their apparent roles in plant development, particularly in the cell cycle, and their observed regulation during AR induction (Ahkami *et al.*, 2014), the HXK-, SnRK1- and T6P-related signalling pathways can be expected to exhibit important functions in mediating sugar signals during the early AR induction and sink establishment phases in cuttings (Fig. 2).

Agulló-Antón *et al.* (2011) observed that cuttings continuously exposed to low light during cold dark storage accumulated extremely high sugar, in particular glucose, levels in the stem base, which was correlated with decreased AR formation even after auxin supply when compared with dark-stored cuttings. In arabidopsis seedlings, glucose inhibited the expression of the *BT2* gene, that has a positive function in auxin response and also depressed the IAA-mediated up- and downregulation of several auxin-responsive genes (Mandadi *et al.*, 2009; Mishra *et al.*, 2009). Considering these findings, Agulló-Antón *et al.* (2011) suggested an inhibitory effect of supra-optimal sugar levels on AR formation via auxin antagonism which may also involve the HXK pathway. Reviewing studies on the relationship between sugar, auxin and plant development, Wang and Ruan (2013) postulated that cross-talk of glucose and auxin controls the cell cycle and the cell expansion in sink tissues via a complex mechanism that involves actions on cyclins and CDKs as well as HXK-mediated gene expression. However, not only antagonistic but also agonistic functions of sugars have been found in relation to auxin signalling (Mishra *et al.*, 2009; Ljung *et al.*, 2015). Furthermore, sugars have been repeatedly reported to stimulate the expression of genes controlling auxin biosynthesis, such as *YUC*, and to alter the distribution of auxin between the shoot and roots (Ljung *et al.*, 2015), suggesting an impact on the auxin homeostasis in the rooting zone of cuttings. There is increasing evidence that TOR is an important linkage point for auxin and nutrient signalling via the modification of protein translation. By sensing both the C and N metabolic status (e.g. via glucose and glutamine) and by responding to auxin, the TOR pathway influences transcriptional and metabolic programmes, while the sensing of N compounds is clearly more complex and far from being understood (Dobrenel *et al.*, 2016; Gent and Forde, 2017). Nevertheless, Deng *et al.* (2017) recently provided evidence that AR formation in arabidopsis seedlings and explants of *Solanum tuberosum* is dependent on TOR function, while chemical inhibition of TOR function strongly alters the expression of genes controlling auxin homeostasis and signalling, and can be partially compensated by overexpression of TIR1.

Sugars may also influence AR formation via modification of the CK pathway. The finding that the shoot meristem in CK receptor mutants of arabidopsis can be partially restored by sucrose-containing medium (Skylar *et al.*, 2011) indicates that sugars and CKs interact in the control of meristem identity (Eveland and Jackson, 2012). In arabidopsis seedlings, glucose affected the transcription of 74 % of CK-regulated genes at the whole-genome level, and genes controlling plant development and the stress response were particularly enriched among the identified genes (Kushwah and Laxmi, 2014). In the same study, high concentrations of glucose reduced the inhibitory effect of high CK concentrations on root growth.

Interestingly, glucose has been shown to act antagonistically on the ET signalling pathway, through a mechanism involving the HXK pathway, and the repression of ABA biosynthetic genes may also be involved in this antagonistic effect (Rolland *et al.*, 2006; Eveland and Jackson, 2012). During the differentiation of epidermal cells in cotyledons of *Vicia narbonensis*, glucose downregulates the expression of ET biosynthetic genes and an *EIN3*-like gene encoding a positive transcriptional regulator of ET signalling (Andriunas *et al.*, 2011).

In summary, there is increasing evidence suggesting that sugars and N metabolites such as amino acids may play a role in AR formation in cuttings as signals and via cross-talk with plant hormone pathways (Fig. 2). However, these relationships are far from being well understood. Functional analyses of genes putatively encoding sugar nutrient sensing components (Fig. 2) and of sugar–hormone cross-talk (Fig. 2) involving dose–response studies of AR formation are necessary to elucidate these relationships.

## GENETIC DIVERSITY IN EXCISION-INDUCED AR FORMATION

Even when functions of genes have not been analysed, correlations between genetic traits and AR phenotypes may help to identify genome sections or even genes that contribute to the genetic diversity of AR formation in the observed group of genotypes. Among eight cultivars of carnation selected from a large, diverse collection of commercial lines, poor-rooting cultivars were characterized by one or several of the following features: a delay in AR initiation, a reduced number of AR primordia or a slow growth rate of ARs (Birlanga *et al.*, 2015). Genome-wide expression profiling and functional changes in the cutting base of two cultivars strongly contrasting in rooting performance revealed that the difference in rooting ability was caused by the delayed activation of formative divisions from cambial cells in the poor-rooting cultivar, which rooting phenotype was rescued by exogenous auxin treatment (Villacorta-Martín *et al.*, 2015). Additional studies confirmed that the differential regulation of endogenous auxin homeostasis in the stem base resulted from differential expression of a specific GH3 protein that limited the accumulation of active auxin in the formative cambial cells of the poor-rooting cultivar (Cano *et al.*, 2018).

Recently, AR formation was studied in a mapping population of poplar established by crossing a hybrid *P. trichocarpa* × *P. deltoides* female with a *P. deltoides* male (Ribeiro *et al.*, 2016). A time-series analysis of transcripts in the parental lines during AR formation revealed that most of the detectable changes occurred in the initial hours after cutting, in association with stress and wounding responses. Transcriptome analyses of individuals with different alleles revealed differentially regulated genes, among which two putative homologues of genes encoding enzymes in the tryptophan biosynthesis pathway were further investigated: *SUPERROOT2* (*SUR2*) and *TRYPTOPHAN SYNTHASE ALPHA CHAIN1* (*TSA1*). *SUR2* encodes a cytochrome P450 enzyme involved in indole glucosinolate biosynthesis, whose inactivation in arabidopsis causes auxin overproduction and an abnormally high number of ARs in the hypocotyl (Barlier *et al.*, 2000). *TSA1* encodes the enzyme that catalyses the conversion of indole-3-glycerolphosphate to indole in the tryptophan biosynthesis pathway (Mano and Nemoto, 2012). The *SUR2* orthologue in poplar undergoes a highly significant reduction in expression during the first hours after cutting in both haplotypes, but its expression remains low at later time points only in poor-rooting individuals. The *TSA1* orthologue also shows reduced expression in individuals with a good rooting ability at later time points, suggesting the utilization of alternative (non-tryptophan) pathways to synthesize IAA (Ribeiro *et al.*, 2016). These results suggest that although poplar genotypes can synthesize auxin through the tryptophan-dependent pathway, the main pathway flux is directed towards the synthesis of indole glucosinolate in the poor-rooting cultivars.

Several clones of *E. globulus* are recalcitrant to AR formation, while *E. grandis* is an easy-to-root species that is widely used in clonal forestry. Through a combination of comparative analyses of gene expression, anatomy and physiology, the possible causes of rooting recalcitrance in *E. globulus* have begun to be elucidated (de Almeida *et al.*, 2015). Auxin levels in the vascular cambium have been measured by immunolocalization fluorescence microscopy in these two species, and higher IAA levels were found in *E. grandis* than in *E. globulus*. Interestingly, the exogenous application of auxin restored the rooting capacity of *E. globulus*. The difference in the expression levels of some auxin biosynthesis and auxin transport genes between *E. grandis* and *E. globulus* might underlie the differential auxin accumulation observed in these species (de Almeida *et al.*, 2015). Additionally, higher expression levels of *TPL* and *IAA12/BDL* in *E. globulus*, whose arabidopsis orthologues co-operate to suppress the expression of auxin-related genes (Szemenyei *et al.*, 2008), suggest a role for this co-repressor module in rooting recalcitrance (de Almeida *et al.*, 2015).

Taken together, the results discussed above indicate that the genetic diversity associated with rooting performance observed both within and between species is, to a large extent, based on the endogenous regulation of active auxin accumulation and auxin responses in the AR source tissues after cutting harvest. Besides these plausible relationships, there is an indication from comparative analysis of cultivars of *Olea europaea*, that *ALTERNATIVE OXIDASE2*, whose encoded protein may function in optimizing photosynthesis and scavenging of reactive oxygen species under stress conditions (Zhang *et al.*, 2010; Xu *et al.*, 2012), might represent a useful marker for the selection of high-rooting olive genotypes at the germplasm level (Macedo *et al.*, 2009; Hedayati *et al.*, 2015).

The genetic variation of AR development and the comparison of genotypes with different abilities to root from stem cuttings has led to the identiﬁcation of quantitative trait loci (QTLs) associated with the number of roots per rooted cutting, for example in two *Eucalyptus* species (Marques *et al.*, 1999), *Pinus elliottii* × *Pinus caribaea* (Shepherd *et al.*, 2006) and poplar (Zhang *et al.*, 2009). Genetic variation and QTL mapping analyses of AR developmental traits have also been reported in crop plants such as *Brassica oleracea* (Oldacres *et al.*, 2005) and *Oryza sativa* (Horii *et al.*, 2006). Since the nutritional status of plants affects AR development, major QTLs have also been identified for adventitious rooting under low-phosphorus conditions in the common bean (Ochoa *et al.*, 2006).

The lack of precise knowledge regarding the location of a QTL, the magnitude of its effects and their biological significance for growth and development are the major limiting factors for the application of QTLs in marker-assisted breeding for AR improvement. However, there are still opportunities for the application of marker-assisted selection, as QTL maps can aid in the selection of long-term strategies in breeding programmes (Marques *et al.*, 1999). The recent developments in high-throughput genotyping platforms, such as SNP (single nucleotide polymorphism) arrays, combined with possibilities for developing mapping populations and the release of genome sequences for crop species propagated by cuttings such as *E. grandis* (Myburg *et al.*, 2014), *Picea abies* (Nystedt *et al.*, 2013), *P. trichocarpa* (Tuskan *et al.*, 2006) and petunia (Bombarely *et al.*, 2016), will allow the exploitation of genetic diversity and the identiﬁcation of QTLs that inﬂuence AR architecture in such plants.

## SYSTEM-ORIENTED CONCEPT: WHAT MAKES THE DIFFERENCE

**Fig. 4.**
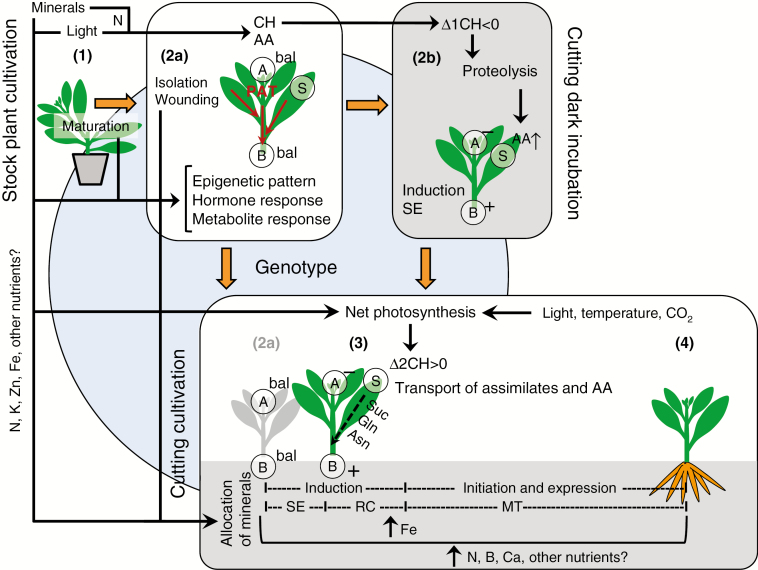
Model of hormonal and metabolic regulation of AR formation in shoot tip cuttings in the context of the plant genotype and selected environmental factors at the stock plant and the cutting levels. The genotype as indicated by the blue circular area provides the genetic background for all responses. The parenthesized number of illustrated cuttings and orange arrows indicate the chronological situations, when cuttings are developing on the stock plant (1), freshly excised (2a) and thereafter either immediately planted and cultivated under light (3) or first incubated in the dark (storage or transport, 2b) before planting (3). A, B and S in circles indicate the shoot apex, stem base and fully developed source leaves as important functional units of the cuttings. Following initially balanced (bal) sink activity between A and B (stage 2a), plus vs. minus signs at B vs. A (stages 2b and 3) indicate the relatively higher sink activity in B compared with A. Black arrows indicate the directions of action. Red arrows indicate the direction of polar auxin transport (PAT). AA, amino acids; CH, carbohydrates; Suc, sucrose; Gln, glutamine; Asn, asparagine; SE, sink establishment; RC, recovery; MT, maintenance. Further explanations are provided in the text.

Formation of ARs in shoot tip cuttings is the result of complex interactions between hormone-related pathways and a complex metabolic response. A possible scenario for the postulated relationships in the context of plant genotypes and selected environmental factors at the stock plant and cutting levels is illustrated in Fig. 4. Fully developed leaves, the shoot apex and the stem base (as the rooting zone) constitute important functional units of such cuttings, acting as source organs, an existing carbohydrate utilization sink and newly developing sink organs, respectively. The genotype (indicated by the overlapping blue background), plant maturation and environmental factors at the stock plant level such as mineral nutrition and light determine the epigenetic constellation and the responsiveness of the cutting to excision at the levels of plant hormones and metabolites. Upon the excision of a cutting (2a in Fig. 4), wounding at the cutting site and isolation from the network of signal and resource fluxes disturb hormonal and metabolic homeostasis. This triggers a hormonal and metabolic response in the stem base, probably modifying the epigenetic constellation of AR source cells (see also Fig. 2). After planting or incubation of the cutting in the dark, auxin and JA activities (Fig. 2) contribute to the induction of ARs and to sink establishment via the activation of sucrolytic enzymes such as invertases (attaining stages 2b or 3 in Fig. 4), thereby enhancing the demand for the subsequent influx of assimilates and co-transported amino acids from the upper shoot. The nutrient levels supplied to stock plants limit the initial abundance of nutrients in the cutting that can be allocated from source tissues to the stem base. The stimulation of AR formation by the high N supply is related to enhanced levels of amino acids at cutting harvest (stage 1 in Fig. 4) and their provision to the stem base (stage 3 in Fig. 4). Furthermore, N-mediated AR formation may involve the stimulation of photosynthesis and sucrose export from source leaves as well as the modification of plant hormone homeostasis and signalling, e.g. via NO (see also Fig. 2). However, the high N supply level leads to a shortage of carbohydrates in the cuttings. Depending on the requirements of the plant species and fertilization of the stock plant, local applications of specific nutrients, such as N, B, Ca and Fe, can stimulate AR formation. Fe may stimulate the division of early meristematic cells, while the phase-specific functions of other nutrients are far from being understood.

The net photosynthesis of cuttings has a strong impact on AR formation via the feeding of sucrose exported from source leaves (stage 3 in Fig. 4) and is dependent on the plant genotype, the acclimation of cuttings to environmental conditions during cutting production and the environmental conditions during the cultivation of the cuttings. Reduced stomatal conductance in response to the interruption of water uptake may inhibit photosynthesis. A depletion of carbohydrates during dark storage (∆1CH <0 at stage 2b in Fig. 4) is more pronounced in N-rich cuttings and reduces the intensity of AR formation under conditions of low photosynthesis in cuttings after planting (low value of ∆2CH at stage 3 in Fig. 4). Subsequently, a prolonged carbohydrate shortage in source leaves limits the influx of carbon via sucrose and co-transported amino acids in the stem base. A progressive depletion of carbohydrates during dark exposure promotes proteolysis in cutting leaves, which can increase the abundance of amino acids in the leaves and rooting zone. When the rate of photosynthesis in cuttings is high, and proteolysis can be reversed, the dark storage of cuttings can advance AR formation via the mobilization of amino acids and the rise in sucrolytic activity in the stem base, enhancing the sink strength relative to the shoot apex, as well as via AR induction (compare stages 2a and 2b in Fig. 4). Auxin transport and enhanced auxin to CK ratios in the stem base most probabely contribute to these dark responses. When the dark-stored cuttings are subsequently planted, high photosynthesis levels allow enhanced transport of sucrose and co-transported amino acids towards the developing ARs.

## FUTURE ASPECTS

Despite advanced research on AR formation in the last decade, important questions remain open for future investigations. These questions include, the functions of new candidate genes, SLs and other underexplored plant hormones; the cross-talk between hormones, particularly that between JA and auxin or between hormones and sugars; the role of miRNAs and genetic and epigenetic control; and the function of limiting mineral elements must be exploited.

Many of the relationships and concepts discussed above are based on data obtained using different plant species, and the results indicate that various physiological bottlenecks may be species dependent. However, the process of AR formation is exploited in diverse horticultural and forestry plants. Therefore, to address this issue, the function of the complete system (cutting in the relevant environment) should be analysed for selected model plants. As a next step, the identified bottlenecks should be tested for relevance to a broader range of (1) cultivars of model species as well as (2) other plant species.

### Analysis of excision-induced AR formation in model plants

Model plants with relevant agricultural, forestry or ecological backgrounds that are suitable for detailed analyses at molecular, physiological and genome levels should be analysed at the following levels.

### Modern biochemical approaches.

Over the last decade, modern methods of chromatography and mass spectrometry have enabled significant advances in tissue- and cell-type-specific analysis and simultaneous profiling of plant hormones (Novak *et al.*, 2017). Lab-on-a-chip or microfluidic technologies using biosensors have emerged in recent years and have been successfully employed to analyse the dynamics of sugar metabolism in intact roots of arabidopsis (reviewed in Stanley *et al.*, 2016). These tools might provide interesting solutions for profiling and localization of hormones and the non-invasive real-time analysis of processes controlling AR formation. Candidate biochemical factors, such as sugars or hormones, can be applied directly or manipulated using products that interfere with their metabolism or transport (Fukui and Hayashi, 2018), while their mode of action may be analysed by monitoring and blocking candidate response pathways. *In vitro* rooting systems are ideal for investigations of sugar–hormone cross-talk (Correa *et al.*, 2005; Birlanga *et al.*, 2015).

#### Sequencing, annotation and assembly of genomes.

The sequencing, assembly and annotation of complete genomes, which are increasingly available for plant species propagated via cuttings, as discussed above, provide the optimal basis for the detection of new candidate genes and analysis of gene function. A virtual transcriptome can be generated *in silico* from the genome. RNA sequencing technology allows the discovery and quantification of RNAs from the entire transcriptome in a single high-throughput sequencing assay (Conesa *et al.*, 2016). For functional analysis, the genome data provide important information about the number of copies of candidate genes in the genome and the structure of genes for the selection and construction of promoters and RNA interference (RNAi) constructs (Sánchez-García *et al.*, 2018).

#### Cell-type-/phase-specific monitoring of the transcriptome, proteome and metabolome.

Considering that root development can be regulated post-transcriptionally at the protein level (Mattei *et al.*, 2013), proteome analysis should complement transcriptome and metabolome approaches. Laser microdissection, immunolocalization or reporter–promoter constructs (de Almeida *et al.*, 2015; Della Rovere *et al.*, 2015) should be used to assign compounds and processes of interest, such as hormones and the expression of genes to particular tissues, cells or even sub-cellular structures. Reporter genes encoding GUS (β-glucuronidase), GFP (green fluorescent protein) or YFP (yellow fluorescent protein) can be fused to the promoter of a target gene, such as the DR5 promoter, used to monitor the auxin response (Voss *et al.*, 2013). However, it must be considered that the DR5 reporting system involves the action of specific ARFs and Aux/IAA proteins, whose expression may be modified during AR formation. In arabidopsis, the DII-Venus sensor has recently been developed as an interesting alternative auxin-sensing system, that is closely linked to the auxin concentration in the tissue, because it uses a rapid maturation form of YFP that is under the direct control of the TIR1/AFB complex as a result of fusion to the auxin-interaction domain DII of the Aux/IAA protein IAA28 (Brunoud *et al.*, 2012; Novak *et al.*, 2017).

#### Mutagenesis, transformation and CRISPR/Cas9.

The function of candidate genes putatively controlling AR formation should be analysed by using mutants such as the transposon insertion line W138 of petunia (Vandenbussche *et al.*, 2016) or plants in which the transcription of target genes has been technically modified. In addition to protocols for *Agrobacterium*-mediated transformation, virus-induced gene silencing and CRISPR/Cas9 (clustered regularly interspaced short palindromic repeats/CRISPR-associated protein 9) technology are available for some plants propagated via cuttings, as recently reported for petunia (Broderick and Jones, 2014; B. Zhang *et al.*, 2016) and poplar (Fan *et al.*, 2015; Shen *et al.*, 2015).

#### Systems biology of the whole response system: cuttings in the environment.

Data determined at different levels, such as the transcriptome or metabolome, with a sufficient temporal and spatial resolution should be integrated into a coherent model of the response and function of the system. Because the processes occurring in the rooting zone of shoot tip cuttings are highly dependent on the function of source leaves and exhibit a competitive relationship with the carbon demand of the shoot apex, which on the other hand may be an auxin source, the whole cutting must be considered as a complex response system. In this context, distinct physiological units of the cutting that present particular functions and respond specifically to environmental factors should be exposed to distinct environmental treatments, and the response at different levels should be integrated into a mechanistic model of the whole cutting. To progress from simple models (e.g. Figs 1–4) to sophisticated models, such as currently being developed to understand the branching of roots (Atkinson *et al.*, 2014), the involvement of additional disciplines, such as mathematics and computer science, is necessary.

#### Genetic mapping and combination with the analysis of complex molecular processes: systems genetics.

After the mapping of QTLs in bi-parental populations using parents with contrasting characteristics or via genome-wide association studies (GWAS) of multiparental populations of different cultivars, genes putatively controlling AR formation may be identified by a comparison of the target genetic intervals or SNPs with the genome sequence, and further functionally analysed. Due to the high complexity of regulatory networks, systems genetics has recently emerged as a sub-field of systems biology that uses expression QTL mapping or co-expression network mapping not only to identify candidate genes underlying the expressed phenotype but also to ascertain the mechanistic context of a gene or gene interaction module (Feltus, 2014).

### Translation of research findings to the broad genetic diversity of crops

To face the genetic diversity, the obtained knowledge of a certain cultivar or species must be translated to other important crop genotypes. Considering the physiological bottlenecks of AR formation in the model systems, cultivars and other species should be tested and categorized into response groups at the physiological level. Ideally, a plant characteristic that determines the respective bottleneck should be used as a measurable marker for the rapid analysis of a large sample number. An example of such an approach to address the N and carbohydrate source limitation of AR formation is the recently developed non-invasive analysis of N_t_, other N fractions and carbohydrates in cuttings by near infrared spectroscopy (Lohr *et al*., 2016, 2017).

## FINAL CONCLUSION

In this review, we considered new findings regarding the hormonal and molecular control of distinct cellular processes of AR formation, and brought them into context with new insights into the role of nutrient and metabolic homeostasis of the whole cutting. Further including a genetic perspective, involving epigenetic factors and facing the complex environment of cuttings, we provided a system-oriented concept on the control of AR formation.

Use of advanced molecular techniques has contributed to further elucidation of processes underlying the important role of auxin in AR formation, as further being linked to ET, JA, CKs and SLs, and interacting with the DNA and chromatin configuration. Wound-responsive *WIND* and *PLT* genes appear to control cell de-differentiation. Specific auxin transporters, ARFs, which are under control of Aux/IAA proteins and miRNAs, as well as GRAS and WOX TFs are highlighted as important factors controlling the determination of root founder cells and the differentiation of ARs. Auxin- and mechanically mediated remodelling of MTs revealed as important process controlling the shift from cell division to cell differentiation, and is further suggested to control the excision-induced anticlinal orientation of cell division in root founder cells.

At the whole-cutting level, AR formation is subjected to the nitrogen and carbohydrate source as controlled by the fertilization and light condition, which determine the surplus of carbohydrates and amino acids in the cutting. Source utilization for AR formation depends on auxin-controlled local activity of sucrolytic enzymes that determines the sink strength of the rooting zone. There, Fe may have a crucial role in ribosome biogenesis as a bottleneck for cell division and differentiation, while the specific functions of other nutrients as well as the signal function of sugars are far from being understood.

Even though substantial progress has been made in the understanding of AR formation, there are still important gaps in our knowledge. To date, most functional studies of genes are restricted to arabidopsis, which does not provide the typical cutting structure used for propagation of many crops. In future, underexplored factors and processes such as *SAUR* genes, sugar and nutrient sensing and hormone–sugar cross-talk should also be exploited in other model plants, and there integrated into coherent models of the response and function of the whole systems. Translation to important horticultural and forestry crops is necessary to provide new knowledge-based breeding and propagation technologies that meet future ecological and economic demands.
